# American Medical Society in Paris

**Published:** 1852-05

**Authors:** 


### AMERICAN MEDICAL SOCIETY IN PARIS.

It will be seen from the following circular that the American physicians in Paris have organized a society for professional improvement. Such a society must prove highly beneficial to the many Americans pursuing their studies in the French capital. We take it for granted that all the medical journals published by their brethren will be forwarded to the society for their use.

### CIRCULAR.

PARIS, (France,) January 10th, 1852.

At a recent meeting of the American Physicians in Paris, an Association was established, whose object is the promotion of Medical Science, This Association, essentially national. is now progressing under the most favorable auspices; it is intended to be permanent in its nature, and is designated the *American Medical Society in Paris.*

Notwithstanding the vast advantages afforded by the French Metropolis, for the study of Medical and Surgical Science, we feel ourselves isolated from our national medical literature, and, therefore, appeal confidently to the conductors of American periodicals and Journals.

We do this with the less hesitation, feeling assured, that it will be not only a medium of improvement to ourselves, but a means of a more general diffusion and just appreciation of American literature.

By order of the Society,

ALEXANDER J. SEMMES, M. D.,

Cor. Sec'y of the American Medical Society in Paris.

N. B. Editors of secular and professional journals will confer a favor by copying the above Circular.

